# Incidence of radiographic humeral bone remodeling after reverse total shoulder arthroplasty and its impact on clinical outcome

**DOI:** 10.1016/j.jsea.2026.100017

**Published:** 2026-04-15

**Authors:** Anthony Timmerman, Jasper De Geyter, Philippe Debeer, Filip Verhaegen

**Affiliations:** a Division of Orthopaedics, University Hospitals Leuven, Leuven, Belgium; b Institute for Orthopaedic Research and Training (IORT), Department of Development and Regeneration, KU Leuven, Leuven, Belgium

**Keywords:** Reverse total shoulder arthroplasty, Radiographic outcomes, Stress shielding, Radiolucent lines, Humeral component

## Abstract

**Background:**

Reverse total shoulder arthroplasty (rTSA) has proven to be a reliable surgical treatment option for a wide variety of shoulder pathologies. Bony remodeling around the humeral component occurs frequently but its impact on clinical outcome remains unclear.

**Methods:**

We retrospectively reviewed 102 patients who underwent primary rTSA between 2008 and 2020. At final follow-up (FU), patients underwent standardized radiographs and clinical examination, including patient-reported outcome measures and the adjusted Constant Murley score (aCMS). Serial radiographs (pre-operative, 6 weeks and ≥2 years post-operative) were evaluated for: bone remodeling (cortical thickness, pedestal formation, spot welds, reactive lines, osteolysis, radiolucent lines) and implant stem parameters (subsidence, size, alignment and filling ratio). Stress shielding was quantitatively defined as a decrease in combined cortical thickness measured at four sites (2 medial [M1 and M2], 2 lateral [L1 and L2]). Subgroup analysis was conducted to determine correlations between the radiographic findings and clinical outcome. The relationship between stress shielding severity and functional outcome was investigated by using percent-based thresholds to explore potential cutoff values.

**Results:**

In 102 patients (mean age 70 years, 71% female, mean FU 65 months), the mean aCMS was 80. Stress shielding occurred in 74%, with combined cortical thickness decreasing from 3.0 to 2.2 mm at final FU; proximal lateral thinning (L1) was most pronounced. A ≥10% cortical thinning threshold at L1 is associated with a lower aCMS (77 vs. 86, *P* = .051). Stem-related parameters, including filling ratio, did not correlate with cortical thinning. Radiographic bone remodeling such as spot welds (36%), reactive lines (49%), and osteolysis (25%) were frequently observed but have no correlation with clinical outcome.

**Discussion:**

This study assessed the long-term radiographic evaluations of humeral bone remodeling after rTSA. The quantitative assessment of cortical thickness showed that the proximal lateral humerus is most affected by stress shielding. A >10% cortical thinning at this site showed a tendency toward inferior clinical outcome, contrasting with prior reports suggesting no functional impact. Other radiographic bone remodeling was common but did not impair function. Our findings support the standardize assessment of cortical resorption with special attention to the region of the proximal lateral humerus during patient FU.

Reverse total shoulder arthroplasty (rTSA) has emerged as an effective solution for managing a wide spectrum of shoulder pathologies with good overall long-term clinical results.^3^^,^^9^^,^^29^ However, no humeral implant can replicate the physiological load pattern on the humeral bone. Therefore, each implant design is associated with a specific load pattern leading to specific adaptive periprosthetic bone remodeling. Furthermore, the semiconstrained design of rTSA imposes higher stresses on the humerus than the native joint, which predisposes humeral bone remodeling like stress shielding, bone resorption and eventual component loosening after rTSA implantation.^27^^,^^31^ Stress shielding is defined by proximal humeral bone loss or bone resorption due to a alterations in loading and strain on the humeral bone after arthroplasty implantation following Wolff's law.^8^

Despite being widely discussed, there is no universally accepted definition or quantification, and many studies simply describe it as “present” or “absent.” More recent, attempts have been made to standardize assessment by grading cortical resorption (0: no resorption; 1: signs of osteopenia; 2: <50% cortical thinning; 3: >50% cortical thinning; 4: complete cortical disappearance), alongside additional remodeling features such as condensation lines or spot welds.^10^^,^^15^ To our knowledge, only 1 previous study measured cortical thickness quantitatively, but merely describing and not correlating it with function.^4^ The effects of implant design, alignment and fixation techniques on bone remodeling are well understood in the context of total hip arthroplasty, where stress shielding is systematically graded using the Engh classification.^6^^,^^7^^,^^12^ However, findings from total hip arthroplasty cannot be directly extrapolated due to the unique anatomical and biomechanical characteristics of the shoulder joint and different implant designs. Therefore, there is growing interest in the interaction between implant characteristics and bone remodeling in rTSA. Long-term radiographic evaluation is crucial to assess features such as stress shielding and implant loosening. Stress shielding is characterized by cortical thinning and osteolysis, while loosening may present as stem subsidence or pedestal formation. These parameters provide critical insights into bone–implant interactions.^30^ Implant design modifications have been explored as strategies to mitigate stress shielding.^22^^,^^23^ Increasing stem size, leading to a higher filling ratio, has shown to increase stress shielding.^16^^,^^24^ Stem length and malalignment — ≥5° varus/valgus alignment — are also noted risk factors.^13^ Additional to positioning and implant design, the fixation technique is also of importance. Brolin et al^4^ noted an increased rate of radiolucent lines and osteolysis for cemented humeral implants in comparison to uncemented humeral implants, where more stress shielding was seen.

However, the clinical implication of these radiographic changes is still up for debate. Stress shieling could lead to late loosening, periprosthetic fracture, and renders revision surgery more challenging. However, these concerns remain theoretical since several studies suggest that stress shielding do not necessarily compromise clinical outcomes, and patients may continue to achieve satisfactory pain relief and shoulder function despite radiographic alterations.^14^^,^^23^^,^^25^ Lafosse et al^17^ demonstrated that functional outcomes remain favorable even after a minimum of 10 years, despite high rate of radiographic changes. In contrast, other reports have highlighted that more advanced bone changes can predispose to loosening and ultimately threaten implant survival.^1^

The aim of this study is to investigate the humeral bone changes after rTSA and apply quantitative measurements of cortical thickness and change over time, using percent-based thresholds rather than relying solely on descriptive reporting of stress shielding. Secondly, we aim to identify technical factors which are associated with these humeral changes. Lastly, we aim to investigate the possible correlation between the radiographical bony changes and clinical outcome. Accordingly, we hypothesize that cortical thinning and other humeral bony changes occur frequently after rTSA and that technical factors are associated with the bony changes. However, we hypothesize that these bony changes are not correlated with the clinical outcome.

## Materials and methods

### Study design and patient characteristics

A retrospective study was conducted at the University hospital Leuven (B3222020000087 – S63831). A total of 440 primary rTSA were implanted between 2008 and 2020 by 2 fellowship trained shoulder surgeons. The inclusion criteria were primary rTSA using the Delta Xtend reverse shoulder system (DePuy Synthes, Raynham, MA, USA) or Trabecular Metal Reverse shoulder system (ZimmerBiomet, Warsaw, IN, USA) with a minimal follow-up (FU) of 2 years. Both systems are long-stem designs relying on diaphyseal intramedullary fixation by different methods. The Delta Xtend stem achieves fixation through a more tapered and fluted design whereas the Zimmer stem demonstrates a more cylindrical distal geometry resulting in higher diaphyseal canal fill. Exclusion criteria were previous septic arthritis of the shoulder and prosthetic surgery for proximal humeral fractures or post-instability arthritis. Ninety-two patients did not meet the inclusion criteria and 90 patients were deceased resulting in a total of 258 patients eligible for inclusion. All patients were invited for a FU visit for clinical and radiological assessment and 102 patients were willing to visit the hospital (response rate 40%). Patient demographics, implant details, and complications were retrieved from the patients' records.

### Clinical assessment

All patients underwent clinical evaluation using the Constant Murley score (CMS), which was adjusted for age and gender leading to the adjusted Constant Murley score (aCMS) and patient-reported outcome measures assessment were recorded during their visit.^5^ Post-operative (POST) pain was recorded using the visual analog scale.

### Radiographical assessment

**Figure 1:**
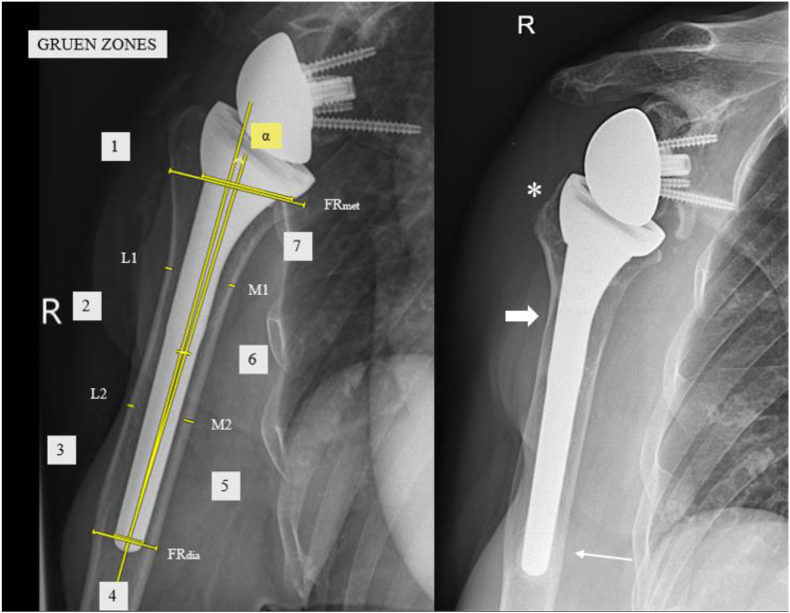
(*Left*) Overview of measurements on radiographs. Gruen zones are depicted in gray squares. Alpha angle (α) is measured between the anatomical axis of the humeral shaft and the long axis of the humeral stem. Filling ratio (FR) at the level of the diaphysis and metaphysis is defined by the quotient of the stem diameter and the humeral diameter at the appropriate level. (*Right*) Follow-up radiograph after 12 years. Asterisk shows greater tuberosity resorption. Big arrow points towards cortical thinning (most pronounced at the level of M1 in this case). Small arrow shows pedestal formation at zone 4.

In total, 3 sequential true glenohumeral anteroposterior radiographs of the shoulder (Grashey view) — taken with the same technical protocol — were available for analysis (maximally 1 year pre-operative [PRE], 6 weeks POST, and minimally 2 years POST [FU]). All radiographs were reviewed by 1 examiner (A.T., orthopedic resident). In order to compare the absolute measurements between radiographs, the magnification factor was calculated on both POST X-rays by using the implant stem size as a calibration marker, matching it with the manufacturer's specifications. The magnification factor of the PRE radiographs was calculated by taking the mean of both POST X-rays. The PRE bone quality/density was assessed using the combined cortical thickness (CCT) in 2 medial and 2 lateral areas of the humerus as described by Tingart et al.^28^ The POST cortical thickness was measured at 4 different locations as described by Nagels et al,^22^ 2 medial and 2 lateral at one-third and two-thirds between the center of the stem proximally and the tip of the stem. Stress shielding was quantitatively defined as a decrease in CCT. The humeral bone remodeling around the stem was further assessed, according to Melis et al^20^^,^^21^ who divided the humerus in 7 zones. These zones were analyzed for the following features: (1) osteopenia, (2) pedestal formation at the tip of the stem, (3) spot welds, (4) reactive lines, (5) osteolysis at the level of the greater tuberosity and the calcar region, and (6) radiolucent lines, which were noted when ≥2 mm.^20^^,^^22^^,^^26^ Loosening was defined as humeral radiolucent lines of ≥2 mm in >3 zones.^11^^,^^20^ Humeral component subsidence was measured by comparing the distance between the most proximal aspect of the greater tuberosity and the tip of the stem at final follow-up minus that at 6 weeks after surgery. An increase in the measured distance showed subsidence of the stem.^2^ Humeral stem alignment was measured by calculating the alpha angle, which is the deviation angle between the anatomical axis of the humeral shaft and the long axis of the humeral stem. More than 5° varus/valgus was defined as malalignment.^13^^,^^24^ Relative stem size was evaluated by using the filling ratio of the metaphysis and diaphysis as described by Schnetzke et al (Fig. 1).^24^

## Statistics

Both quantitative and a qualitative analysis were performed. Categorical variables are presented as counts and proportions. Normality was assessed using the Shapiro-Wilk test for numerical data. Normally distributed data were represented by means and standard deviations (SDs), and non-normally distributed data by medians and interquartile ranges. Longitudinal changes in cortical thickness at the four measurement sites and the combined cortical thickness were analyzed using paired comparisons between the serial radiographs. Exploratory analyses were conducted to evaluate potential cutoff values that might discriminate clinical outcome groups. Correlation between radiographical bony changes and clinical outcomes were assessed using Spearman rank correlation coefficient. Comparison between groups was made using the Mann-Whitney *U* test for continuous outcomes and the Chi-square test for categorical outcomes. A *P* value lower than 0.05 was considered statistically significant. Statistical analysis was performed using Statistical Analysis System (SAS) software (version 9.4) of the SAS System for Windows.

## Results

### Demographics and patient cohort

A total of 102 patients were included in the study, with a mean age of 70 years (SD 8 years, ranging from 51 to 85 years). The general data are listed in Table I. The mean visual analog scale score was 1.38 (SD 2.07) at rest and 2.63 (SD 2.65) during activity at the latest FU. The mean CMS was 68 (SD 17, range 14-98) at FU, while the aCMS score was 80 (SD 21, range 16-118).

**Table I:** Demographics: characteristics of patients and surgical technique.

Characteristics	Patient cohort
Overall, N	102
Age, mean (range), years	70 (51-85)
Sex: male/female, n(%)	30 (29%)/72 (71%)
BMI: mean + - SD	28 +- 6
Follow-up: mean (range), months	65 (29-158)
Type of rTSA: Delta Xtend/Zimmer, %	63/37
Type of fixation: cemented/uncemented, %	23/77

*BMI*, body mass index; *SD*, standard deviation; *rTSA*, reverse total shoulder arthroplasty.

## Radiographic outcomes

**Figure 2:**
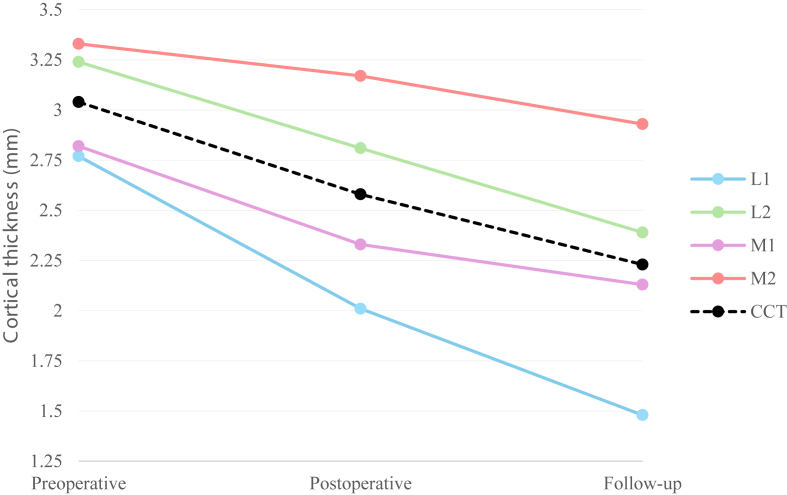
Overall tendency of the evolution of the cortical thickness (mm) from the preoperative (PRE) radiograph, the post-operative (POST) X-ray and the X-ray at last follow-up (FU).

**Figure 3:**
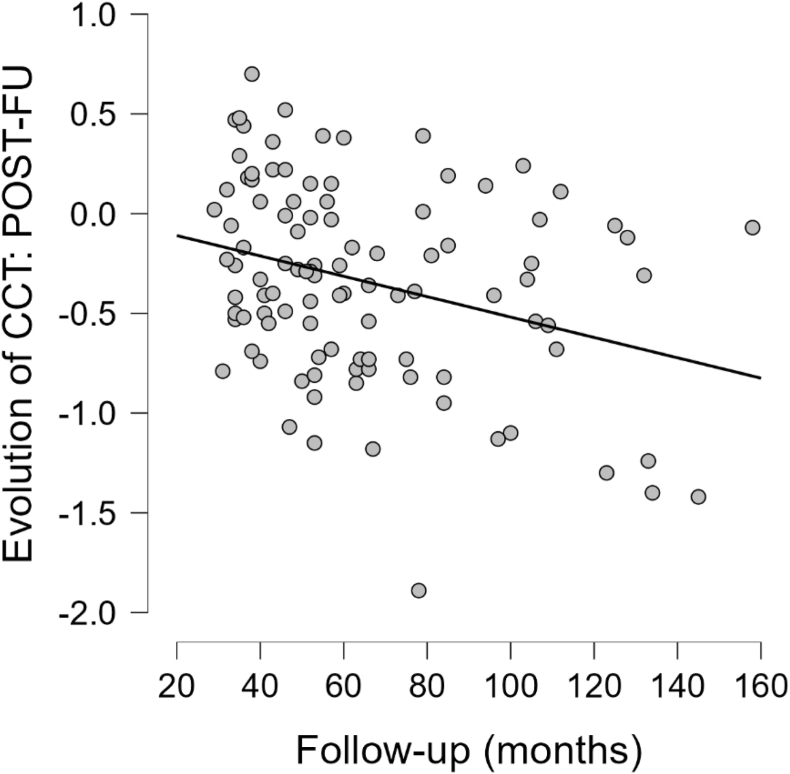
Cortical thickness diminishes over time (Spearman rho = −0.309, *P* value = .002).

Stress shielding was present in 74% (75/102) of the patients. Overall, the mean CCT decreased from 3.0 mm to 2.2 mm at final FU, which is a 27% relative reduction of CCT (Table II, Fig. 2). Longer FU resulted in more decrease in CCT (*P* = .003, Fig. 3). POST proximal cortical thinning was more pronounced (mean change = −0.48 mm) compared to distal regions (−0.24). The most pronounced remodeling was observed at the proximal lateral cortex (L1, Gruen zone 2). Spot welds were identified in 37 patients (36%), most commonly in zone 3 and zone 6.

**Table II:** Mean difference (in millimeters) of cortex measurements between radiographs.

Time point/comparison	L1	L2	M1	M2	CCT
Pre-operative (PRE)	2,8	3,2	2,8	3,3	3,0
Post-operative (POST)	2,0	2,8	2,3	3,2	2,6
Follow-up (FU)	1,5	2,4	2,1	2,9	2,2
Δ PRE-FU	−1,3	−0,9	−0,7	−0,4	−0,8
Δ POST- FU	−0,5	−0,4	−0,2	−0,2	−0,4
%Δ POST-FU	26,4%	14,9%	8,6%	7,6%	13,6%

*CCT*, combined cortical thickness.

Mean subsidence, was 1.7 mm (range 0-6 mm). Three patients had a substantial subsidence of >5 mm. At POST measurements there were no malaligned stems. Over time, 2 patients had a malaligned stem >5° varus/valgus at FU. Pedestal formation was seen in only 4 patients. Osteolysis was observed in 26% of patients at the greater tuberosity and 26% at the calcar region (Fig. 1). More combined cortical thinning was seen in the presence of pedestal formation (ρ = −0.267, *P* = .008) and osteopenia (ρ = −0.252, *P* = .12). Six percent of the patients had notable radiolucent lines ≥2 mm but none of these were in ≥3 zones.

### Technical factors

The Zimmer stem demonstrated a significantly higher diaphyseal filling ratio compared to the Delta Xtend stem (0.59 vs. 0.50, *P* < .001); however, this difference did not translate into increased CCT reduction (−0.25 vs. −0.39 mm, *P* = .234), nor was it associated with other radiographic bony changes. Stem diameter and filling ratio were further analyzed in relation to cortical remodeling. Grouping by stem size (10-16 mm) did not reveal significant differences in cortical thinning at the level of L1 (ρ = 0.039, *P* = .697). Similarly, the diaphyseal filling ratio at FU showed no significant correlation with cortical thickness change at L1 (ρ = 0.083, *P* = .407). While not statistically significant, there was a trend toward increased osteolysis at the greater tuberosity (*P* = .062) and calcar region (*P* = .111) with larger stem size. Neither initial POST stem alignment (*P* = .390) nor the use of cement (*P* = .475) influenced cortical thinning at L1.

### Clinical outcome

As the L1 site was identified as the most frequent location of stress shielding it was chosen as the reference site for percent-based thresholds to explore potential cutoff values for defining stress shielding severity and its relationship with aCMS (Table III). Cutoff values of ≥10% reduction in CCT at L1 showed a meaningful separation in aCMS. Patients with ≥10% of L1 cortical thinning had a lower mean aCMS (77) compared to those with <10% thinning (86), approaching statistical significance (*P* = .051). No higher thresholds demonstrated significant differences in clinical outcome. Patients with cemented rTSA had better functional outcome than uncemented rTSA (aCMS 77 vs. 88, *P* = .022). Other radiographic bony changes did not correlate with the functional outcome (Table IV).

**Table III:** Cut-off values for stress shielding and parameters of stem stability.

Thresholds	No (A)	Yes (B)	Mean aCMS A	Mean aCMS B	*P* value∗
≥0% L1 change	13	89	89	78	.071
≥10% L1 change	25	77	86	77	.051
≥20% L1 change	39	63	83	77	.163
≥30% L1 change	56	46	83	76	.190
≥50% L1 change	82	20	80	79	.853
≥75% L1 change	100	2	79	84	.875
Subsidence ≥5 mm	99	3	80	74	.596
Malalignment ≥5°	100	2	80	65	.197
Signs of loosening	96	6	81	69	.223
Cemented rTSA	79	23	77	88	**.022**

*aCMS*, adjusted Constant Murley score; *rTSA*, reverse total shoulder arthroplasty.

Bold indicates statistical significant result (*P* < .05).

∗ Mann-Whitney *U* test.

**Table IV:** Prevalence of radiographic bone changes by Gruen zones and correlation with function.

Radiographic features	Present	Zone 1	Zone 2	Zone 3	Zone 4	Zone 5	Zone 6	Zone 7	aCMS present group	aCMS absent group	Spearman ρ (rho)	*P* value
Osteopenia	57%	21%	66%	50%	47%	7%	21%	50%	79	79	0.011	.912
Pedestal formation	4%	/	/	/	100%	/	/	/	74	80	−0.022	.832
Spot welds	36%	0%	14%	38%	3%	19%	41%	0%	78	80	−0.078	.438
Reactive lines	49%	0%	0%	22%	94%	22%	0%	0%	80	78	−0.005	.965
Greater tuberosity osteolysis	25%	100%	/	/	/	/	/	/	79	80	−0.030	.770
Calcar osteolysis	26%	/	/	/	/	/	/	100%	78	80	−0.046	.646
At risk for loosening	6%	33%	33%	33%	50%	50%	17%	17%	69	81	−0,126	.222

*aCMS*, adjusted Constant Murley score.

## Discussion

This study provides a detailed radiographic evaluation of the humeral component and surrounding humeral bone in rTSA, with a mean FU exceeding five years. Stress shielding was seen in 74% of our patients, which is similar to the 84% described by Giol et al.^10^ The proximal lateral humerus was most affected by stress shielding, as evidenced by pronounced cortical thinning in this region, aligning with mechanical theories that proximal regions endure lower stress due to load transfer to the implanted stem.^22^ While most studies categorize stress shielding as “present” or “absent,” we used quantitative cortical thickness measurements between the immediate POST and FU radiographs. The amount of cortical thinning that we identified — a decrease of −26% for L1, -15% for L2, -9% for M1, and -8% for M2 — was somewhat greater than the literature available literature. Our longer mean FU of 65 months in comparison to 35 months likely accounts for this difference.^4^ Interestingly, a cutoff of ≥10% L1 thinning shows a tendency to inferior clinical outcome scores reaching the MCID for the CMS.^18^ This contrasts with previous reports that stress shielding does not compromise function. A potential explanation is that our site-specific quantitative approach is more sensitive for detection of subtle changes in cortical bone. Moreover, the L1 zone may be particularly relevant, as it might have an influence on the quality and functionality of the posterior cuff insertion hence affecting shoulder function. Considering these findings, we support to standardize assessment by grading cortical resorption with a special attention to the region of the proximal lateral humerus when evaluating clinically relevant bone remodeling.^10^^,^^15^ We found no association between stem diameter, filling ratio or alignment and the degree of cortical thinning. Although the Zimmer stem demonstrated a significantly higher filling ratio compared to the Delta Xtend stem, which is to be expected based on its more cylindrical distal geometry, this did not translate into increased cortical thinning or other radiographic bone changes. This contrasts with earlier studies in shoulder arthroplasty where larger stems and higher diaphyseal filling ratios have been linked to more pronounced bone remodeling. Schnetzke et al^24^ reported that both metaphyseal and diaphyseal filling ratios were independent predictors of stress shielding, suggesting that oversized implants compromise load distribution between bone and implant and accelerate bone loss; however, this study was performed on short-stem implants. Raiss et al^23^ demonstrated that malalignment, particularly exceeding 5° varus or valgus, predisposes to increased stress shielding. Our results diverge from these findings, likely due to differences in implant design as our cohort entirely exists of long stemmed rTSA, which minimizes the risk of malalignment and therefore off-axis loading. Although long stems are theoretically associated with more proximal stress shielding due to distal load transfer, the uniformity of stem sizing and consistent diaphyseal fixation in our cohort may have reduced interpatient variability, thereby masking detectable correlations between filling ratio and bone loss.

Subsidence was modest overall (mean 1.7 mm) and only 3 cases exceeded ≥5 mm, which did not demonstrate inferior function, suggesting that minor migration represents benign adaptation rather than mechanical failure. Interestingly, cemented stems achieved better clinical outcomes than uncemented stems, aligning with the findings of Mazaleyrat et al.^19^ A potential explanation is that cement may buffer micromotion and redistribute loads more evenly along the stem. Other radiographic changes such as spot welds, reactive lines, pedestal formation, and tuberosity osteolysis were frequently observed in our series, occurring in up to half of the cases. Importantly, none of these findings were associated with impaired clinical outcomes. This is consistent with prior reports indicating that most humeral bone adaptations after rTSA are radiological phenomena without clinical consequences.^30^ Our findings reinforce this perspective, showing that radiological changes alone should not be equated with clinical failure. On the contrary, certain features such as spot welds may indicate adaptive remodeling and stable bone-implant integration, as described by Nagels et al.^22^ Taken together, our results support the view that most radiographic changes after rTSA are widespread but often benign, highlighting the need to distinguish between pathological loosening and adaptive bone remodeling. This distinction is clinically important, as overemphasis on radiographic findings without functional correlation may lead to unnecessary concern or overtreatment.

While our study provides valuable insights, several limitations should be acknowledged. First, the lack of PRE questionnaires limits our ability to evaluate baseline patient-reported outcomes comprehensively. Second, the response rate of 40% may introduce selection bias, although it is worth noting that this rate is reasonable given the geriatric nature of the population, the duration of FU and the geographic challenges posed by the university hospital's wide referral area. Third, radiographic measurements were performed by a single observer, which may limit reproducibility despite standardized measurement protocols. Finally, our cohort was limited to long-stemmed humeral implants, and therefore the results may not be generalizable to other rTSA designs.

## Conclusion

This study provides a comprehensive radiographic evaluations of humeral bone remodeling after stemmed design rTSA, with a large cohort and long-term FU. Quantitative analysis of cortical thickness revealed pronounced proximal lateral thinning, with a >10% cutoff approaching clinical relevance for functional outcome. Contrary to theoretical expectations, stem size and filling ratio were not predictive of cortical thinning. Other radiographic changes were common but not clinically consequential. These findings suggest that future studies should standardize assessments by grading cortical resorption for stress shielding, particularly at the proximal lateral cortex, instead of a qualitative assessment.

## Disclaimers:

Funding: No funding was disclosed by the authors.

Conflicts of interest: Philippe Debeer: The Institute for Orthopaedic Research and Training (IORT), Department of Development and Regeneration, KU Leuven, Division of Orthopaedics, University Hospitals Leuven reports research grant funding from the following organizations, which are not related to the topic of this work: charitable donations from Zimmer-Biomet (EDW-ZIMMER-O2030), the PROSPEROS I (2014TC16RFCB046) and PROSPEROS II project (ZL3C150100-401) funded by the Interreg VA Flanders–The Netherlands program. None of these outside sources or funders were involved in the study design, data collection, data analysis, or the preparation or editing of the manuscript. Philippe Debeer reports consultancy agreement with Materialise. Philippe Debeer holds a Hip and Shoulder Research Johnson educational chair. These relationships have no bias on this work and did not inappropriately influence this study. Filip Verhaegen: The Institute for Orthopaedic Research and Training (IORT), Department of Development and Regeneration, KU Leuven, Division of Orthopaedics, University Hospitals Leuven reports research grant funding from the following organizations, which are not related to the topic of this work: charitable donations from Zimmer-Biomet (EDW-ZIMMER-O2030), the PROSPEROS I (2014TC16RFCB046) and PROSPEROS II project (ZL3C150100-401) funded by the Interreg VA Flanders–The Netherlands program. None of these outside sources or funders were involved in the study design, data collection, data analysis, or the preparation or editing of the manuscript. Filip Verhaegen reports consultancy agreements with ZimmerBiomet and Exactech Inc. Filip Verhaegen holds a Hip and Shoulder Research Johnson educational chair. These relationships have no bias on this work and did not inappropriately influence this study. Any additional authors, their immediate family, and any research foundation with which they are affiliated have not received any financial payments or other benefits from any commercial entity related to the subject of this article.

Patient consent: Obtained.
